# Reference frames in allocentric representations are invariant across static and active encoding

**DOI:** 10.3389/fpsyg.2013.00565

**Published:** 2013-08-28

**Authors:** Edgar Chan, Oliver Baumann, Mark A. Bellgrove, Jason B. Mattingley

**Affiliations:** ^1^Queensland Brain Institute, The University of QueenslandSt. Lucia, QLD, Australia; ^2^School of Psychology, The University of QueenslandSt. Lucia, QLD, Australia; ^3^School of Psychology and Psychiatry, Monash UniversityClayton, VIC, Australia

**Keywords:** spatial cognition, reference frames, object-location memory, navigation, allocentric

## Abstract

An influential model of spatial memory—the so-called *reference systems* account—proposes that relationships between objects are biased by salient axes (“frames of reference”) provided by environmental cues, such as the geometry of a room. In this study, we sought to examine the extent to which a salient environmental feature influences the formation of spatial memories when learning occurs via a single, static viewpoint and via active navigation, where information has to be integrated across multiple viewpoints. In our study, participants learned the spatial layout of an object array that was arranged with respect to a prominent environmental feature within a virtual arena. Location memory was tested using judgments of relative direction. Experiment 1A employed a design similar to previous studies whereby learning of object-location information occurred from a single, static viewpoint. Consistent with previous studies, spatial judgments were significantly more accurate when made from an orientation that was *aligned*, as opposed to *misaligned*, with the salient environmental feature. In Experiment 1B, a fresh group of participants learned the same object-location information through active exploration, which required integration of spatial information over time from a ground-level perspective. As in Experiment 1A, object-location information was organized around the salient environmental cue. Taken together, the findings suggest that the learning condition (static vs. active) does not affect the reference system employed to encode object-location information. Spatial reference systems appear to be a ubiquitous property of spatial representations, and might serve to reduce the cognitive demands of spatial processing.

## Introduction

Successful navigation in novel environments requires accurate encoding and retrieval of object-location information, both in terms of the positions of salient landmarks in relation to oneself and also their positions with respect to one another. To successfully navigate around an unfamiliar city, for example, one needs to identify the locations of street signs, intersections and buildings with respect to one's current position (“egocentric representations”), as well as the positions of prominent distant landmarks with respect to each other (“allocentric representations”). A key question in recent navigation research has been how allocentric or “object-centered” representations are organized within long-term memory. According to an account proposed by McNamara and colleagues, object-to-object spatial relationships are organized with respect to a specified reference system (Shelton and McNamara, 2001; Mou et al., 2004). The directions or “axes” used for the reference system are biased by factors such as egocentric viewpoint or salient environmental cues, such as the geometry of a room, or the layout of buildings in a city. The purpose of the present study was to examine the extent to which these biases play a role in the formation of spatial memories under differing learning conditions.

**Figure 1:**
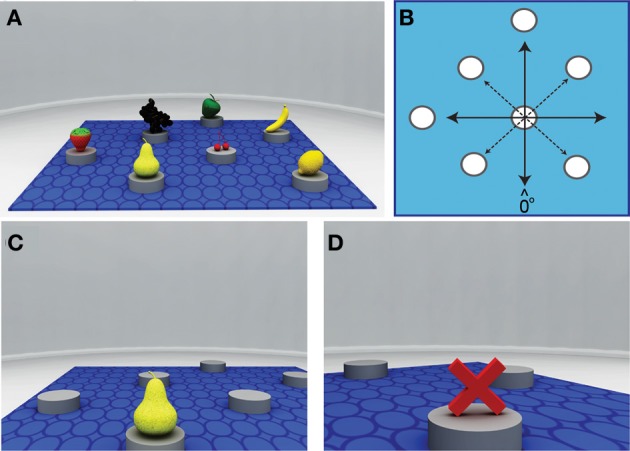
**Schematic and local views of the virtual arena used during the learning phase. (A)** Survey view of the arena showing the object array and the square mat (in blue) that provided the extrinsic frame of reference. **(B)** The spatial arrangement of objects in the array (white circles). Inter-object relationships are thought to be encoded preferentially with respect to coordinates *aligned* with the sides of the square mat (solid lines), as opposed to *misaligned* (dashed lines). **(C)** In Experiment 1B, when participants arrived at a correct location, the probed target object (e.g., a pear) appeared immediately above the placeholder. **(D)** When participants arrived at an incorrect location, a red cross appeared above the placeholder.

Evidence that object locations are organized around reference systems has been derived principally from experiments that have employed static arrays of items on a table-top or in a room. In a typical task, participants are asked to study and remember the spatial arrangement of an object array from a single viewpoint. Within the array, a prominent environmental feature (e.g., a square-shaped mat) or structure (e.g., the room geometry) is included to emphasize, perceptually, one or more spatial axes. Assessment of participants' spatial memory for object locations is probed using a so-called “Judgment of Relative Direction” (JRD) task, which requires participants to imagine themselves standing at the position of one object, facing a different object, and then to point to the location of a third object. Studies that have used this approach have typically shown that participants are faster and more accurate when performing the JRD task for imagined orientations that are *aligned* (i.e., parallel or orthogonal) with the perceptually salient structure, than for orientations that are *misaligned* (see Figures 1A,B; Shelton and McNamara, 2001; Mou and McNamara, 2002; Valiquette et al., 2003, 2007; Valiquette and McNamara, 2007). Such observations of superior performance for aligned vs. misaligned orientations are thought to reflect the fact that inter-object spatial relationships are represented in memory with respect to salient geometric features available in the environment. On this account, spatial relationships that are aligned with the adopted frame of reference are represented explicitly, thus, supporting faster and more accurate judgments of relative direction, whereas relationships that are not aligned with the frame of reference must be inferred, leading to a cost in judgments of relative direction (Shelton and McNamara, 2001).

Several previous studies have focused on examining the different types of environmental cue that can act as a frame of reference for organizing spatial memories. Intrinsic array properties, such as the physical arrangement of objects in rows and columns (Mou and McNamara, 2002), environmental properties such as room geometry or other salient features (Shelton and McNamara, 2001; McNamara et al., 2003; Kelly and McNamara, 2008; Kelly et al., 2013), as well as the egocentric viewpoint of the observer and verbal cueing (Greenauer and Waller, 2008), can all bias the structure of the internal spatial reference system. Recent studies have shown that an established reference system at one point in time can be exploited to organize new spatial information within the same environment at a later point in time (Greenauer and Waller, 2010; Kelly and McNamara, 2010). Furthermore, reference systems have been shown to play a role in spatial recognition (e.g., Mou et al., 2008) and spatial updating processes (e.g., Zhang et al., 2011).

Most studies that have examined the influence of environmental reference frames have employed static arrays of items on a table-top or in a small-scale room, such that spatial information is acquired via a survey (i.e., aerial or map-like) perspective [though see McNamara et al. (2003)]. However, in a series of studies, Shelton and colleagues showed that encoding via survey or route (i.e., dynamic, ground-level) perspectives can result in distinct behavioral and neural retrieval patterns (Shelton and Gabrieli, 2002; Shelton and McNamara, 2004; Shelton and Pippitt, 2007). Whereas survey or global perspectives provide explicit information about the overall spatial structure of an environment, object-to-object spatial relationships must be inferred in the route or ground-level perspective via spatial updating processes (Montello, 1993; Tversky, 2005).

In addition to the possible effects of perspective type, the number of viewpoints available to participants might also influence subsequent spatial representations. Learning of spatial arrays in studies of reference frames is generally conducted via passive viewing of an environment from a limited number of viewpoints (e.g., Shelton and McNamara, 2001; Kelly and McNamara, 2008; Kelly et al., 2013) or restricted movement (e.g., McNamara et al., 2003; Tlauka et al., 2011). In contrast, spatial learning in everyday circumstances often involves active exploration of an environment with an unrestricted number of viewpoints. For example, when navigating around a university campus, one might approach different landmarks from different viewpoints depending on which classes are to be attended on a given day. Active viewing or moving within an environment can result in superior spatial performance compared with passive viewing (e.g., either viewing sequential visual displays or being physically moved through an environment; e.g., Christou and Bulthoff, 1999; Jurgens et al., 1999; Sun et al., 2004; Waller and Greenauer, 2007). These findings have been attributed to an increase in an individual's control over incoming sensory information during active exploration, and to the availability of proprioceptive and vestibular cues. Active exploration generally increases the amount of visual information available to the observer, via exploratory head and eye movements, and this in turn might provide a richer representation of the learned environment (Evans and Pezdek, 1980).

To our knowledge, only one study has investigated the influence of reference systems using a paradigm that allowed free-movement during the encoding of spatial locations (Valiquette et al., 2003, Experiments 2 and 3). Participants in that study were required to remember an object array presented within an enclosed rectangular room by picking up and replacing each object in its designated location over repeated trials. Participants' retrieval performance indicated that their spatial representations were orientation-dependent, biased by environmental cues, despite experiencing the array from multiple views and headings. This indicated that free exploration may not be sufficient to build up an orientation-free (unbiased) representation of the environment. However, spatial learning in the study by Valiquette and colleagues occurred within an environment in which participants were able to survey the entire object-array without the need for navigation. In contrast, two studies that have examined spatial alignment effects in large, familiar environments in which participants were not able to apprehend the whole environment from one viewpoint, have found contradictory results. A study by Sholl (1987), which looked at the role of cardinal directions for pointing accuracy within a familiar university campus, found that judgments of directions for landmarks did not differ as a function of the participant's heading. That is, internal spatial representations did not appear to be biased by any particular cardinal direction. A more recent study by Frankenstein et al. (2012) found evidence that familiar large-scale environments can be represented within a single, north-oriented reference frame, similar to one acquired from a map. Pointing accuracy of long-term residents of a German town toward highly familiar local landmarks was best when the participants were oriented north, and performance worsened with increasing deviation from north. As yet, however, it remains unclear whether the same finding would also apply when salient environmental cues are present in the environment.

Against this background, the aim of our experiment was to examine and compare the influence of salient environmental cues on object-location memory under two distinct encoding conditions. Experiment 1A employed a design similar to that of previous studies in which learning of object-location information occurred from a single, static viewpoint that allowed participants to view the entire layout from an aerial perspective. This was to assess the validity and effectiveness of our novel virtual environment, by replicating the classic alignment effect (Shelton and McNamara, 2001; Mou et al., 2004). In Experiment 1B, participants learned the same object-location information through active navigation from a ground-level perspective, such that object-to-object relationships had to be integrated over multiple viewpoints. We compared differences in spatial retrieval performance across different imagined viewpoints as a measure of orientation-dependence. We tested whether allocentric spatial representations are biased by available environmental cues and, if so, how these are affected by differences in encoding conditions.

## Experiment 1A

In Experiment 1A we examined whether a salient environmental feature can bias the reference system of an object array within a novel virtual environment using a single static snapshot. Participants were shown an image of a circular arena containing seven distinct target objects, and were asked to remember their locations (see Figure 1A). A square mat was positioned on the floor of the arena to provide an “extrinsic” frame of reference (i.e., a coordinate axis external to the object array). Spatial knowledge of the array was tested using a JRD task similar to that employed in previous studies (e.g., Shelton and McNamara, 2001). Consistent with previous findings (Shelton and McNamara, 2001; Mou and McNamara, 2002; Valiquette et al., 2003, 2007; Valiquette and McNamara, 2007), we hypothesized that judgments of object location immediately after learning should reflect a spatial representation organized with respect to the extrinsic frame of reference. Specifically, we predicted that performance would be better for imagined orientations aligned with the coordinate axis of the mat than for misaligned orientations.

### Methods

#### Participants

Sixteen students (8 males) from The University of Queensland participated in the experiment, either for course credit or monetary compensation. All were naïve to the study aims and experimental paradigm prior to participation.

#### Stimuli and design

The virtual environment was created using the Blender open source 3D content creation suite (The Blender Foundation, Amsterdam, The Netherlands) and presented on a 21.5-inch LCD monitor. Two arrays of 7 objects were arranged in a symmetrical configuration so that they could be perceptually grouped vertically, horizontally or diagonally (see Figure 1B). Common fruits were chosen as objects as they are visually distinctive and contain no intrinsic spatial relationship. The array of objects was arranged on top of a square blue mat (equivalent to 324 m^2^ in virtual space). The mat served as a salient environmental feature, providing an extrinsic frame of reference by highlighting the vertical and horizontal alignment of objects within the array, parallel to the orthogonal axis of the mat (Figure 1B). The object array and mat were placed within a circular arena (radius = 23 m; area = ~1662 m^2^) that was void of any other distinct visual cues. For the *learning phase* of the task, a snapshot of the array was taken from a position 30 m from the center of the arena and 10 m above the floor. This provided the clearest view of the virtual environment, the object array, and the square mat. The snapshot was taken so that it was aligned with the sides of the mat.

Participants' memory of the object array after learning was assessed using a standard JRD task that required participants to point in the direction of a target object from an imagined position and orientation within the arena. Each trial commenced with a written instruction that provided participants with their position and orientation within the arena, in addition to an object to point toward. Thus, for example, a typical instruction might read: “*Imagine you are standing at the pear, facing the grapes. Point to the cherries.*” The primary independent variable was the *imagined heading*, defined by the specified standing and facing positions (in the previous example, the participant should imagine facing the grapes from the perspective of the pear). The imagined heading parallel with the participants' learning perspective was defined as 0°, and the remaining seven possible orientations were labeled 45–315° (each 45° apart) in a counter-clockwise fashion. Pointing responses were recorded using a single hand-grip joystick. The main dependent measure was the angular error (i.e., the absolute difference in the recorded pointing direction and the actual direction of the target object). Trials were presented using Presentation software (Neurobehavioral Systems; http://www.neurobs.com). Participants completed 80 JRD trials during each retrieval phase, 10 for each orientation. Object frequency and pointing direction were approximately equal across orientations. Trial order was randomized for each participant and completed in 2 blocks with a short break between them.

#### Procedure

***Learning phase***. Prior to the beginning of the experiment, participants familiarized themselves with the virtual environment by freely exploring the arena from a ground-level perspective using a joystick. The object-array was not present during this period. Once familiarized, participants were shown an aerial snapshot of the arena containing the object array (as shown in Figure 1A), and instructed to study and remember the locations of the objects with respect to each other. The snapshot was shown for 30 s, after which participants' knowledge of the array was probed by asking them to imagine standing inside the arena from the learned perspective with their eyes closed, and to point to the objects one by one, using a laser pointer in their right hand, as requested by the experimenter. Pointing responses were monitored online to ensure that the remembered locations were correct. Participants were then shown the same snapshot of the array repeatedly for 30 s periods until they were able to point to each of the seven object locations without hesitation. On average, participants required two or three such exposures (i.e., 60–90 s of viewing) before proceeding to the testing phase.

***Testing phase***. Written instructions for the test trials were displayed on the same LCD monitor as was employed in the learning phase, and responses were made using the joystick. Prior to the test phase, participants completed 3–5 practice trials of the JRD task using prominent landmarks around the university campus (e.g., “From where you are sitting, point to the Great Court”). Each test trial consisted of a fixation cross for 2.5 s, followed by the JRD instruction screen which indicated the imagined position, facing direction and target object for that trial. The instructions remained on the screen until participants moved the joystick in the desired direction and pressed a button to indicate their response. Participants were asked to respond as accurately as possible.

### Results and discussion

**Figure 2:**
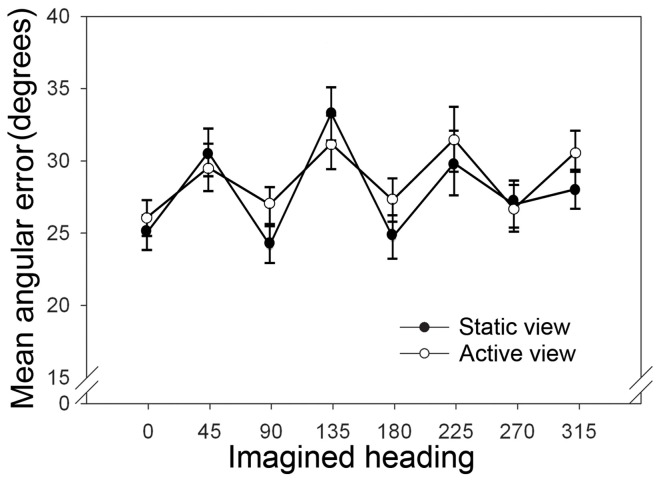
**Results of the judgement of relative direction (JRD) task in Experiments 1A,B**. Mean angular error in degrees, shown as a function of imagined heading for Experiment 1A (static view—filled symbols) and Experiment 1B (active view—open symbols). Error bars represent ±1 normalized within-subjects error of the mean (Cousineau, 2005).

Absolute pointing error on the JRD task was calculated as the unsigned difference between the participant's response and the correct direction of the target object. Trials in which pointing error was >90° were removed from the analyses (3%). Figure 2 (filled symbols) shows the mean pointing error plotted as a function of imagined heading.

**Figure 3:**
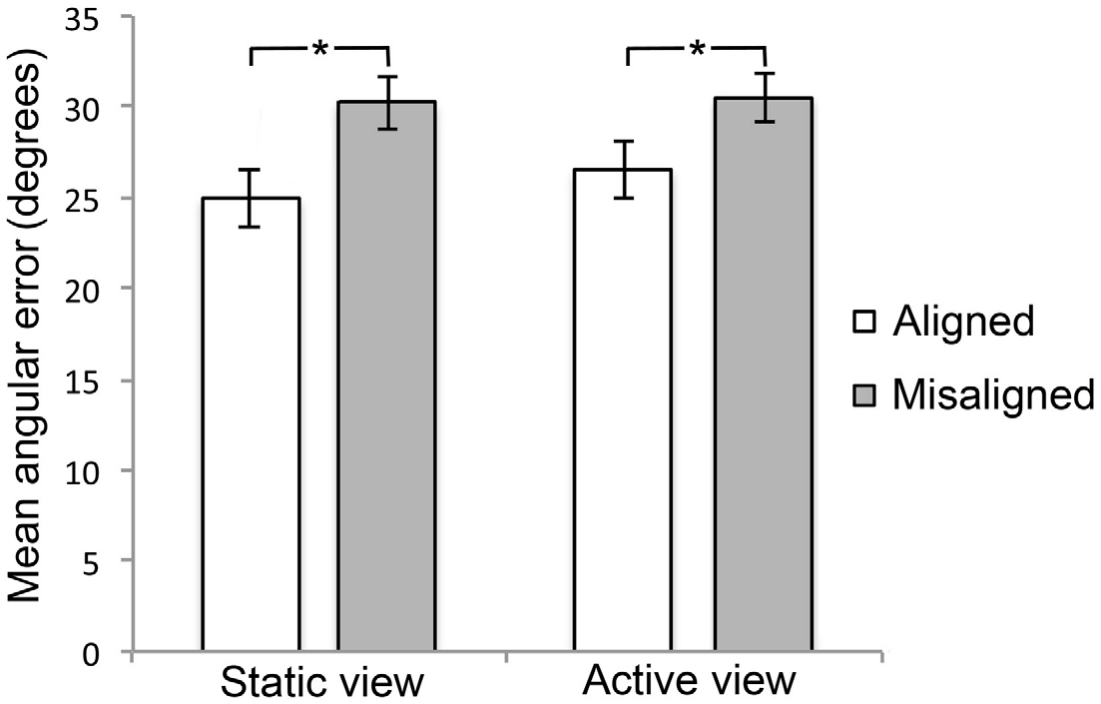
**Mean performance for the aligned and misaligned headings between the two learning conditions**. Mean angular error in degrees for the aligned (white bar) and misaligned (gray bar) headings in Experiment 1A (static view) and Experiment 1B (active view). The brackets represent statistically significant differences between the two types of imagined headings (^*^p = 0.05).

Consistent with previous findings, participants' pointing accuracy was better for imagined headings aligned with the orthogonal axis of the mat (0°, 90°, 180°, 270°) than for imagined headings that were misaligned (45°, 135°, 225°, 315°). These observations were confirmed statistically. The 8 levels of imagined heading were collapsed into a more theoretically meaningful factor of *alignment* with two levels: aligned (means of 0°, 90°, 180°, 270°) and misaligned (means of 45°, 135°, 225°, 315°; see Figure 3). A paired-samples *t*-test comparing the aligned vs. misaligned conditions revealed a significant difference [*t*_(15)_ = 4.41, *p* < 0.01]. We also tested whether retrieval performance for the learning orientation (i.e., 0°) was different from the other three aligned orientations, but found no significant effect. In addition to conventional null-hypothesis significance testing, we conducted Gallistel's (2009) Bayesian analysis to estimate the odds in favor of the null hypothesis: that retrieval performance is not different between the learning orientation and the other three aligned orientations that were not experienced during learning [see Kelly et al. (2013) for an example]. Analyses yielded odds of 4.43:1 (weight: 0.65) for 0–90°, 4.20:1 (weight: 0.62) for 0–180°, and 3.96:1 (weight: 0.60) for 0–270°. These odds and weights are considered substantial (c.f. Gallistel, 2009) and in favor of the hypothesis that retrieval performance is equivalent between the learning orientation and the other three aligned orientations.

The findings of Experiment 1A suggest that a salient environmental feature can bias the spatial reference system used to represent an object array presented from a single, static viewpoint, consistent with previous investigations (Shelton and McNamara, 2001; Mou and McNamara, 2002; Valiquette et al., 2003, 2007; Valiquette and McNamara, 2007).

## Experiment 1B

In Experiment 1B, we examined whether changing the learning condition of object-location information would alter the bias exerted by the salient environmental cue. Specifically, we investigated whether free-exploration from a ground-level perspective would lead to the development of an orientation-free spatial representation. The environmental layout was identical to that of Experiment 1A. Critically, however, the learning phase of the task was manipulated so that the relevant spatial layout of environmental objects could only be apprehended from the ground-level perspective through active navigation and integration of information accumulated over time. If it is a ubiquitous property of spatial memory that object-locations are referenced to, and biased by, the presence of a salient environmental boundary, there should be a significant orientation-dependent alignment effect similar to that found in Experiment 1A. If, however, free exploration involves the integration of multiple viewpoints and the development of an orientation-free representation, there should be no significant effect of alignment on JRD performance.

### Methods

#### Participants

Twenty students (10 males) from The University of Queensland participated in the experiment for monetary compensation. All participants were naïve to the study aims and experimental paradigm prior to participation.

#### Stimuli and design

The virtual environment was identical to that of Experiment 1A, with the exception that a set of circular gray placeholders (radius = 0.7 m, height = 0.5 m) now marked the locations of the target objects (fruits). Participants explored the arena from a ground-level perspective at a viewing height of 1.8 m, and navigated the arena using a handheld joystick (max velocity = 3 m/s). In successive trials, participants searched for the location of a cued target object by trial-and-error (see Figures 1C,D). When the correct location was reached, the target object appeared above the placeholder. When an incorrect location was reached, a red cross appeared instead, prompting the participant to continue searching.

#### Procedure

***Learning phase***. Participants were instructed at the beginning of the experiment that they had to navigate within a virtual environment to find and remember the locations of hidden fruits. Participants were instructed to pay particular attention to the object locations in relation to each other, as this information would later be tested. Participants practiced the task in a different virtual arena until they understood the task instructions.

Each trial began with a 1500 ms display which showed the target object to be discovered for that trial. Participants were then transported to the start location (18 m from the center of the arena), and had to locate the object as quickly as possible. Arriving at a gray placeholder either revealed the target object (a correct location) or a red cross (an incorrect location). The trial continued until the participant found the correct location, after which the next trial was initiated. Each of the seven target objects was cued in a random order without replacement, before any object was cued again. The learning phase was terminated when participants had correctly located each target object within the array, without navigating to an incorrect placeholder, for two of the previous three trials in which that object was cued.

***Testing phase***. The testing phase was the same as that for Experiment 1A.

### Results and discussion

Participants took an average of 27.5 trials (range: 20–47 trials) to reach criterion during the learning phase. On average, participants were cued to each target object four times (range: 3–7 times). Correlation analysis showed that there was no significant relationship between the number of trials participants completed in the learning phase and the magnitude of pointing error in the JRD task (*p* > 0.1). Thus, the number of trials spent learning object locations within the arena was not related to the magnitude of the alignment effect.

Figure 2 shows the mean pointing error for the JRD task (open symbols) plotted as a function of imagined heading. Trials in which pointing error was >90° were removed from the analyses (5%). The pattern of results was similar to that observed in Experiment 1A. Participants were more accurate for imagined headings aligned with the coordinate axis of the extrinsic frame of reference (0°, 90°, 180°, 270°) than for imagined headings that were misaligned (45°, 135°, 225°, 315°). Statistical analysis confirmed these observations. A paired-samples *t*-test comparing the mean angular error for aligned (0°, 90°, 180°, 270°) vs. misaligned (45°, 135°, 225°, 315°) headings revealed a significant difference between the two conditions [*t*_(19)_ = 2.911, *p* = 0.039; see Figure 3]. We also tested whether retrieval performance for the initial starting orientation (i.e., 0°) differed from the other three aligned orientations, but found no significant effect. As in Experiment 1A, analyses using Gallistel's (2009) Bayesian approach revealed odds and weights in support of performance equivalence between the initial starting orientation and the other three aligned orientations [4.26:1 (weight: 0.63) for 0–90°, 4.06:1 (weight: 0.61) for 0–180°, and 5.21:1 (weight: 0.72) for 0–270°].

To determine whether differences between the learning conditions affected JRD performance, we compared the results of Experiments 1A and 1B directly. The objects, their spatial locations, and the mat were the same across these two experiments; the only difference was the manner of object array encoding (static aerial viewpoint vs. active ground-level viewpoint). A repeated-measures ANOVA with the between-subjects factor of experiment (1A vs. 1B) and the within-subjects factor of alignment (aligned, misaligned) was performed on mean angular error. There was a significant main effect of alignment [*F*_(1, 34)_ = 24.341, *p* < 0.001, η^2^ = 0.417], but no significant main effect of experiment (*p* > 0.1) and no interaction between these factors (*p* > 0.1)^1^. A further analysis was performed in which the eight imagined headings were entered as separate levels within the ANOVA, to test for potential differences in performance for specific orientations. This analysis also failed to yield any significant effects involving the factor of experiment. In addition to conventional null-hypothesis significance testing, we also conducted Gallistel's (2009) Bayesian analysis on the aligned and misaligned headings between the two experiments, to estimate the odds in favor of the null hypothesis. Analyses yielded “substantial” odds of 3.97:1 (weight: 0.60) and 5.11:1 (weight: 0.71) for the aligned and misaligned conditions, respectively, in favor of the null hypothesis.

The findings of Experiment 1B suggest that salient environmental features continue to bias allocentric representations of object locations when learning occurs through active navigation. Furthermore, our findings suggest that the reference system used in allocentric spatial representations is invariant across static and active learning conditions within the same environment.

## General discussion

A growing body of research has found evidence to suggest that object-location representations are organized around reference frames determined by salient environmental cues. Most previous studies that have investigated this phenomenon have been conducted via passive viewing of small environmental arrays from a limited number of viewpoints (e.g., Shelton and McNamara, 2001; Kelly and McNamara, 2008; Kelly et al., 2013) or with restricted movement (e.g., McNamara et al., 2003; Tlauka et al., 2011). As yet, there is little evidence to indicate the extent to which a salient environmental feature influences the formation of spatial memories when spatial learning occurs via free and active navigation; a scenario more in line with real-world navigation. Moreover, the potential effect of different learning conditions on the development of spatial reference systems has not been directly investigated within the same environment.

Consistent with previous studies (Shelton and McNamara, 2001; Mou and McNamara, 2002; Valiquette et al., 2003, 2007; Valiquette and McNamara, 2007), Experiment 1A showed that a salient environmental feature biases the spatial reference system used to represent an object array encoded from a single static viewpoint. Participants' pointing accuracy was better for imagined headings aligned than misaligned to the salient environmental feature. Furthermore, retrieval performance for the learned orientation (i.e., 0°) was equivalent to that of the other three aligned orientations (Kelly et al., 2013). To our knowledge, this is the first demonstration that alignment effects can be evoked by a salient geometric cue through learning of a purely 2D display. Other studies have conducted the learning of object-location information using real-world environments such as table-tops and rooms, and then examined spatial learning using computer displays. Although one other study has used a virtual environment to display object-location information (Kelly and McNamara, 2008), the authors used a fully-immersive head-mounted display which allowed physical turning and walking to mimic real-world exploration. Our findings mirror those of other studies that used real-world or quasi-real world environments, demonstrating that real or 3D structures are not necessary for inferring environmental reference systems for object-location representations. We have shown that 2D environments are most likely processed in a similar way to real-world environments, as both contain information that is within the immediate surrounding and can be apprehended from a single viewpoint.

More strikingly, in Experiment 1B we found that a salient environmental feature can bias allocentric representations of object locations even when learning occurs through active navigation. These results are not consistent with previous findings that suggest free-exploration should lead to an orientation-free spatial representation (Sholl, 1987) or a single, north-oriented representation (Frankenstein et al., 2012). Prior to this, one other notable study by McNamara et al. (2003) has investigated the influence of reference systems within a large, real-world space in which object-location information had to be integrated over time. Participants were asked to remember the locations of specific objects encountered during a guided walk along a path that was either aligned or misaligned with a frame of reference prescribed by a large rectangular building in the center of a park. Results showed that participants guided along the aligned path used the rectangular building as a frame of reference for the organization of object-location information, whereas those guided along the misaligned path did not use the rectangular building. However, in that study the path traversed by participants during learning was pre-determined, and participants' body-orientation was restricted. Findings from our study suggest that the rectangular building may have biased spatial representations irrespective of movement restrictions.

As both Experiments 1A and 1B were conducted within the same environment, and with the same dependent variables, we were able to directly compare performance between the two experiments to test for any effect of learning condition on object-location representations. Surprisingly, we found that the pattern of object-location retrieval from memory was not statistically different for situations in which learning occurred via unconstrained active navigation (Experiment 1B) and via a single, global-snapshot (Experiment 1A). Bayesian analyses confirmed that the odds were substantially in favor of the null hypothesis, that learning condition (static vs. active) does not significantly influence spatial retrieval performance. This suggests that the biases induced by a salient environmental cue in internal spatial representations are ubiquitous, at least across static and active spatial learning conditions. This finding contradicts previous studies that have found differences in performance for active compared with passive spatial learning (e.g., Christou and Bulthoff, 1999; Jurgens et al., 1999; Sun et al., 2004; Waller and Greenauer, 2007). One possibility for this difference may be that the environments used in those studies did not contain environmental cues that were salient enough to consistently bias internal reference systems across different learning modalities. Our study shows that if a salient environmental feature exists in the environment to provide an external frame of reference, it will bias internal spatial representations irrespective of learning conditions. Such landmark-centered reference systems may serve to reduce cognitive load and increase efficiency, but come also at the cost of potential spatial inaccuracies (e.g., poorer spatial judgments of relative direction). Tversky (2005) has proposed that these systematic errors are unlikely to have any practical implications for accurate navigation. However, this has not been investigated empirically and would certainly be an interesting avenue to explore in future. For example, it would be interesting to examine whether errors in judgments of relative direction during imagined heading translate to similar actual navigation inaccuracies during movement toward target object locations.

In conclusion, our findings demonstrate that salient environmental features can systematically bias internal spatial representations across different learning conditions. Our study emphasizes the importance of spatial reference systems in spatial cognition. Recent fMRI studies have found evidence to suggest behavioral alignment effects are associated with distinct patterns of neural activity (Xiao et al., 2010; Chan et al., 2013). Future research should focus on how this empirically robust spatial alignment effect translates to real-world navigation behavior.

### Conflict of interest statement

The authors declare that the research was conducted in the absence of any commercial or financial relationships that could be construed as a potential conflict of interest.

## Footnotes

^1^Given previous reports of gender differences in spatial abilities [see reviews by Voyer et al. (1995); Coluccia and Louse (2004)], we re-ran the repeated-measures ANOVA adding gender (male, female) as an additional between-subjects factor. There was no significant main effect or interaction involving gender (*p* > 0.1).
